# The impact of plasmonic electrodes on the photocarrier extraction of inverted organic bulk heterojunction solar cells

**DOI:** 10.1007/s00339-023-06492-6

**Published:** 2023-03-02

**Authors:** Florian Kolb, Mirella El Gemayel, Imran Khan, Jakub Dostalek, Roman Trattnig, Christian Sommer, Emil J. W. List-Kratochvil

**Affiliations:** 1 Institute of Surface Technologies and Photonics, Joanneum Research Forschungsges. mbH, Franz-Pichler-Straße 30, 8160 Weiz, Austria; 2 AIT-Austrian Institute of Technology GmbH, BioSensor Technologies, Konrad-Lorenz-Straße 24, 3430 Tulln, Austria; 3 FZU-Institute of Physics, Czech Academy of Sciences, Na Slovance, 182 21 Prague, Czech Republic; 4 Institut für Physik, Institut für Chemie & IRIS Adlershof, Humboldt-Universität zu Berlin, Zum Großen Windkanal 2, 12489 Berlin, Germany; 5 Helmholtz-Zentrum Berlin für Materialien und Energie GmbH, Hahn-Meitner-Platz 1, 14109 Berlin, Germany

**Keywords:** Organic solar cells, Electrode patterning, Optical enhancement, Surface plasmons, Dielectric waveguide modes, Charge carrier extraction

## Abstract

Nano-patterning the semiconducting photoactive layer/back electrode interface of organic photovoltaic devices is a widely accepted approach to enhance the power conversion efficiency through the exploitation of numerous photonic and plasmonic effects. Yet, nano-patterning the semiconductor/metal interface leads to intertwined effects that impact the optical as well as the electrical characteristic of solar cells. In this work we aim to disentangle the optical and electrical effects of a nano-structured semiconductor/metal interface on the device performance. For this, we use an inverted bulk heterojunction P3HT:PCBM solar cell structure, where the nano-patterned photoactive layer/back electrode interface is realized by patterning the active layer with sinusoidal grating profiles bearing a periodicity of 300 nm or 400 nm through imprint lithography while varying the photoactive layer thickness (L_*PAL*_) between 90 and 400 nm. The optical and electrical device characteristics of nano-patterned solar cells are compared to the characteristics of control devices, featuring a planar photoactive layer/back electrode interface. We find that patterned solar cells show for an enhanced photocurrent generation for a L_*PAL*_ above 284 nm, which is not observed when using thinner active layer thicknesses. Simulating the optical characteristic of planar and patterned devices through a finite-difference time-domain approach proves for an increased light absorption in presence of a patterned electrode interface, originating from the excitation of propagating surface plasmon and dielectric waveguide modes. Evaluation of the external quantum efficiency characteristic and the voltage dependent charge extraction characteristics of fabricated planar and patterned solar cells reveals, however, that the increased photocurrents of patterned devices do not stem from an optical enhancement but from an improved charge carrier extraction efficiency in the space charge limited extraction regime. Presented findings clearly demonstrate that the improved charge extraction efficiency of patterned solar cells is linked to the periodic surface corrugation of the (back) electrode interface.

**Supplementary Information:**

The online version contains supplementary material available at 10.1007/s00339-023-06492-6.

## Introduction

Organic solar cells (OSCs) offer many distinct advantages as they allow for the utilization of cost-effective, low temperature and large area and continuous fabrication techniques on flexible substrates, making OSCs highly attractive for numerous applications [1–4]. Yet, OSCs suffer from low power conversion efficiencies (PCEs), compared to inorganic and hybrid solar cells, which slightly diminishes their attractiveness [5–7]. However, using non-fullerene polymer-based acceptors led to recent advances in the PCE (> 18%) that put OSCs back in the race again [8, 9]. In addition, tremendous efforts have been devoted to improve the performance of the photoactive layer and thereby the solar cell PCE using light trapping concepts [10–12]. In particular, periodically patterning the interface between the active layer and the metal back electrode is considered as a highly promising candidate to enhance the solar cell absorption efficiency, even beyond the well-known Yablonovitch limit [13, 14]. The presence of a patterned active layer/(metal) electrode interface allows for the resonant coupling of incident light to propagating surface plasmon (PSP) and dielectric waveguide (WG) modes. Excitation of these optical modes significantly improves the spatial confinement of the incident optical fields within the solar cell, translating into an increased absorption probability of impinging photons [11, 15–18]. The impact of the geometrical pattern parameters, such as pattern profile, pattern periodicity or pattern height on the characteristic of the observed absorption enhancements has been extensively covered in literature [19–25]. Due to their simple fabrication, typically 2D pattern structures (e.g., linear grating profiles with the corrugation in one direction along the surface) are used to investigate the mechanisms of enhancing the absorption characteristics of patterned OSC. This pattern design, however, intrinsically limits the magnitude of the observed PSP-related absorption enhancements as only light in TM polarization can couple to a PSP mode. In contrast, utilizing 3D pattern structures (e.g., cross-grating profiles with the corrugation varying in both orthogonal directions along the surface) when patterning the active layer/back electrode interface has been shown to excite PSPs for TM- and TE-polarized light [26, 27]. Moreover, advanced pattern structures such as multi-diffractive grating couplers composed of various spatial pattern periodicities were proposed that allow to spectrally broaden the observed absorption enhancements through the simultaneous excitation of multiple optical light-coupling modes [28–31].

Aside from improving the OSC absorption characteristics in the visible and near-infrared wavelength range, patterning the active layer/back electrode interface additionally impacts the electrical device parameters, since the patterning modifies the spatial photocurrent generation profile and the carrier extraction processes. Utilizing a patterned electrode interface has been shown to decrease the device series resistance that was attributed to an increased interface area, improving both the device fill factor (FF) and short circuit current density (${J}_{SC}$), as compared to devices with a planar electrode interface [27, 32–34]. However, increasing the absolute interface area can also increase the total number of surface defects acting as recombination centers for the light induced photocarriers [35, 36]. Yet, the impact of a patterned device electrode on the OSC electrical characteristic is not just limited to charge collection mechanisms at the active layer/electrode interface. Compared to solar cells with a planar back electrode surface geometry, the excitation of PSPs induces a strong spatial confinement of the optical fields in close vicinity to the patterned electrode interface, shifting the spatial photocurrent generation from the (transparent) front electrode to the back electrode [37–39]. The PSP-related modification of the spatial photocurrent generation profile when patterning the back electrode interface, therefore, alters the mean path length light-induced photocarriers need to cover until extraction at the respective device electrodes. For photocarriers harvested at the front and back electrode, the mean extraction path length is increased and reduced, respectively. To minimize non-geminate recombination at solar cells, the extraction path length needs to be shorter than the diffusion/drift length of the light-induced photocarriers [40, 41]. Therefore, a variation of the photocarrier extraction path length could significantly affect the impact of non-geminate recombination on the harvested photocurrents yields. Recently, the PSP-induced variation of the photocarrier mean extraction path length in presence of a patterned back electrode interface was shown to mitigate non-geminate recombination caused by the introduction of space charges [38]. This mechanism, however, was only observed at inverted solar cells (i.e., patterned anode interface) but not at devices bearing a standard device architecture (i.e., patterned cathode interface) [38]. Although this report meticulously examines the space charge limited photocurrent extraction characteristic of standard and inverted P3HT:PCBM solar cells, it does not specifically address the impact of a patterned back electrode interface at different charge extraction regimes or when varying the applied pattern period of the utilized 2D pattern structure [38].

In this work, we thoroughly discuss for two distinct pattern periodicities (i.e., Λ = 300 nm, 400 nm) how patterning the back electrode affects the extraction of light generated photocarriers at different extraction regimes. Focusing on inverted P3HT:PCBM bulk heterojunction OSCs, different charge extraction regimes are realized by varying the photoactive layer thickness (L_*PAL*_) in the range between 90 and 400 nm. For an in-depth understanding of the experimental OSC performance, finite-different-time-domain (FDTD) simulations are performed in dependence of L_*PAL*_ and with respect to the surface profile of the active layer/back electrode interface (i.e., planar or patterned). The simulation findings are further validated by monitoring the generation of the spectral dependent photocurrents through the experimental external quantum efficiency (EQE) characteristics of fabricated solar cells. Our optical and electro-optical examinations are complemented by a detailed investigation of the voltage and irradiance dependent charge extraction characteristic, obtained from fabricated planar and Λ = 300 nm, 400 nm patterned solar cells.

## Experimental

### Materials

Poly(3-hexylthiophene-2,5-diyl) (P3HT) with a regio-regularity between 91 and 94% and a molecular weight (M_*W*_) of 50 kDa < M_*W*_ < 70 kDa was obtained from Rieke Metals LLC (Referencer #4002-E). The polymer polyethyleneimine (PEI), the fullerene derivate [6,6]-phenyl-C61-butyric acid methyl ester (PCBM) and all solvents (i.e., chlorobenzene (CB), 2-Butanol) were purchased from SIGMA-ALDRICH and used as received. Pre-patterned Glass/ITO substrates with a specified nominal sheet resistance of 15 **Ω**/**□** were obtained from Kintec Company.

### Fabrication of imprinting stamps

Patterning of the solar cell back electrode (anode) was achieved through a thermal-assisted nanoimprint lithography approach, where a soft mold is used for transferring the nano-sized surface relief profiles from a master template into a polymer layer at elevated temperatures (i.e., soft embossing technique). The master template used at our investigations was prepared by UV-laser lithography and a detailed description of the procedure and the process parameters has been given elsewhere [30]. For the soft mold, the Sylgard^™^ 184 elastomer kit was purchased from Dow and the polydimethylsiloxane (PDMS) and a curing agent was mixed at a ratio of 10: 1. In the next step, the mixture was poured over the master template and weak vacuum conditions (*p* ~ 10 mbar) were applied to remove air entrapments. The mold was cured at a temperature of ~ 70 °C for a duration of 3 h, afterwards allowed to cool down to room temperature and carefully detached from the master template.

### Device fabrication and imprinting procedure

For fabricating ITO/PEI/P3HT:PCBM/MoO_*X*_/Ag bulk heterojunction (BHJ) solar cells, the P3HT and PCBM compounds were mixed at a weight ratio of 1:0.75 (P3HT:PCBM), then dissolved in chlorobenzene (CB), heated to a temperature of 75 °C and stirred for 3 h. Subsequently, the solution cooled down to room temperature and was continuously stirred for another 12 h to ensure a complete dissolution of the solid compounds. PEI was solubilized in 2-Butanol at a concentration of 0.15 g/l by continuously stirring for 12 h at room temperature. Pre-patterned glass/ITO substrates were thoroughly cleaned by subsequently immersing the substrates in a diluted Hellmanex^®^ (2%), an acetone and an isopropanol ultrasonication bath at a temperature of 50, 45 and 40 °C, respectively, for a duration of 15 min each. Successive fabrication steps were conducted in a glove box under an inert (Ar) atmosphere, where the respective oxygen and water levels were kept below 10 ppm. The PEI solution was spun on the glass/ITO substrates at a rate of 1500 rpm for 5 s followed by a rate of 4000 rpm for 20 s. To remove solvent residuals, samples were transferred to a hot-plate and annealed at a temperature of 105 °C for 10 min. Next, P3HT:PCBM solutions were spun at a rate of 1200 rpm for 40 s. The P3HT:PCBM layer thickness was varied by adjusting the P3HT:PCBM concentration between 20 and 60 g/l. Obtained P3HT:PCBM layer thicknesses were 90 ± 10 nm (20 g/l), 175 ± 12 nm (30 g/l), 284 ± 11 nm (40 g/l) and 394 ± 16 nm (60 g/l) as evaluated by an atomic force microscope. For imprinting the pattern profile, the PDMS mold was placed on the P3HT:PCBM layer immediately after spin-coating and then thermally annealed at a temperature of 135 °C for 20 min. Utilized pattern profiles (i.e., linear 2D sinusoidal grating profile) had a pattern periodicity of 300 nm and 400 nm, respectively, while the obtained imprint depth (i.e., peak-to-trench distance) was about 30 nm for both pattern periodicities (see Figure S1). To ensure for a conformal coating of the materials comprising the device back electrode (i.e., anode) on the P3HT:PCBM layer top surface, Mo_*X*_ and Ag materials were subsequently deposited through thermal evaporation at a base pressure < 1 × 10^–6^ mbar and at a deposition rate of 0.1 Å/s (MoO_*X*_) and 5 Å/s (Ag), respectively. The thickness of the MoO_*X*_ and the Ag layers was fixed to 10 nm and 100 nm for all examined solar cell samples.

### Simulation setup

The optical characteristics as well as the spatial distribution of the optical near-field of the investigated glass/ITO/P3HT:PCBM/MoO_*X*_/Ag solar cells were simulated through a finite-difference time-domain (FDTD) approach, implemented by Ansys Canada Ltd. The refractive indices of the glass (BK7) [42], ITO [43] and Ag [44] layer materials used in the simulations were taken from literature, while the optical constants of MoO_*X*_ and the P3HT:PCBM blend were obtained through variable angle spectroscopic ellipsometry measurements (see Figure S2). In the simulations, Cartesian coordinates were used with the *x*- and *y*-axis in plane of the layers and with the *z*-axis perpendicular to the layer surface. The 2D grating profile of fabricated patterned solar cells at interfaces between the P3HT:PCBM, MoO_*X*_ and Ag layers was approximated using the height function h(*x*) = A sin(2π/Λ *x*) with Λ as the pattern periodicity and A as the pattern amplitude. The amplitude A was set to a 15 nm throughout the simulations (i.e., peak-to-trench distance = 30 nm). A light source was placed 200 nm below the glass/ITO interface to generate plane waves in the spectral range between 350 and 850 nm that propagate along the *z*-axis (i.e., normally incident on the pattern structure). Periodic boundary conditions were used in x-direction while perfectly matched layers (PMLs) were placed 500 nm above the Ag layer and 500 nm below the glass/ITO layer interface. The width of the simulation unit cell in the lateral direction was defined as two periods of Λ. The optical near-field spatial distribution and the reflectance (*R*) of the planar and corrugated geometries was obtained through a set of field monitors. The absorption characteristic (Abs) of investigated solar cell stacks was acquired through the relation Abs [%] = 100 – *R* [%].

### Ellipsometry measurements

The refractive indices of the P3HT:PCBM and the MoO_*X*_ layer materials were evaluated through variable angle spectroscopic ellipsometry (VASE) measurements (J.A. Woolam Co., Inc.). Obtained ellipsometry measurement data was further analyzed by the WVASE software package (J.A. Woolam Co., Inc.). A detailed description of the process parameters used at the deposition of the P3HT:PCBM and the MoO_*X*_ films and of the refractive index modelling procedure is given in the Supporting Information.

### Topography characterization

An atomic force microscope (AFM; Nanoscope V, Veeco Digital Instruments), operated in tapping mode, was used to assess the photoactive layer thickness (L_*PAL*_) and for monitoring the imprint quality (i.e., the pattern amplitude and the pattern periodicity) of patterned solar cells.

### Solar cell performance and external quantum efficiency characterization

For evaluating the solar cell performance, the voltage dependent current density (J–V) characteristics of fabricated planar and patterned solar cells was acquired with a Keithley 2400 source measurement unit at a AM1.5G calibrated solar simulator (ATLAS Material Testing), equipped with HMI lamp (HMI 1200W/DXS, OSRAM GmbH) that provided an irradiance of 100 mW/cm^2^. The external quantum efficiency (EQE) of fabricated solar cells was obtained by a custom-built EQE unit, where a Xe light source in combination with a monochromator (LOT QuantumDesign, MSH 150) allows to spectrally tune light in the range between 350 and 800 nm. A set of optical lenses was used to ensure a minimal angular divergence of the light beams normally incident on the device samples. A spectral broad-band polarizer was used to acquire the EQE characteristics in dependence of the polarization state of incident light. The EQE unit was additionally equipped with a Lock-In Amplifier (Stanford Research Systems, SR830 DSP) and a light chopper, operated at a frequency of 465 Hz, to enhance the measurement sensitivity and to limit the measurement noise. During the evaluation of the device performance and the EQE characteristics, fabricated solar cells were mounted in a measurement cell filled with Ar-gas to avoid device degradation caused by the presence of water and oxygen [45–47].

## Results and discussion

**Fig. 1:**
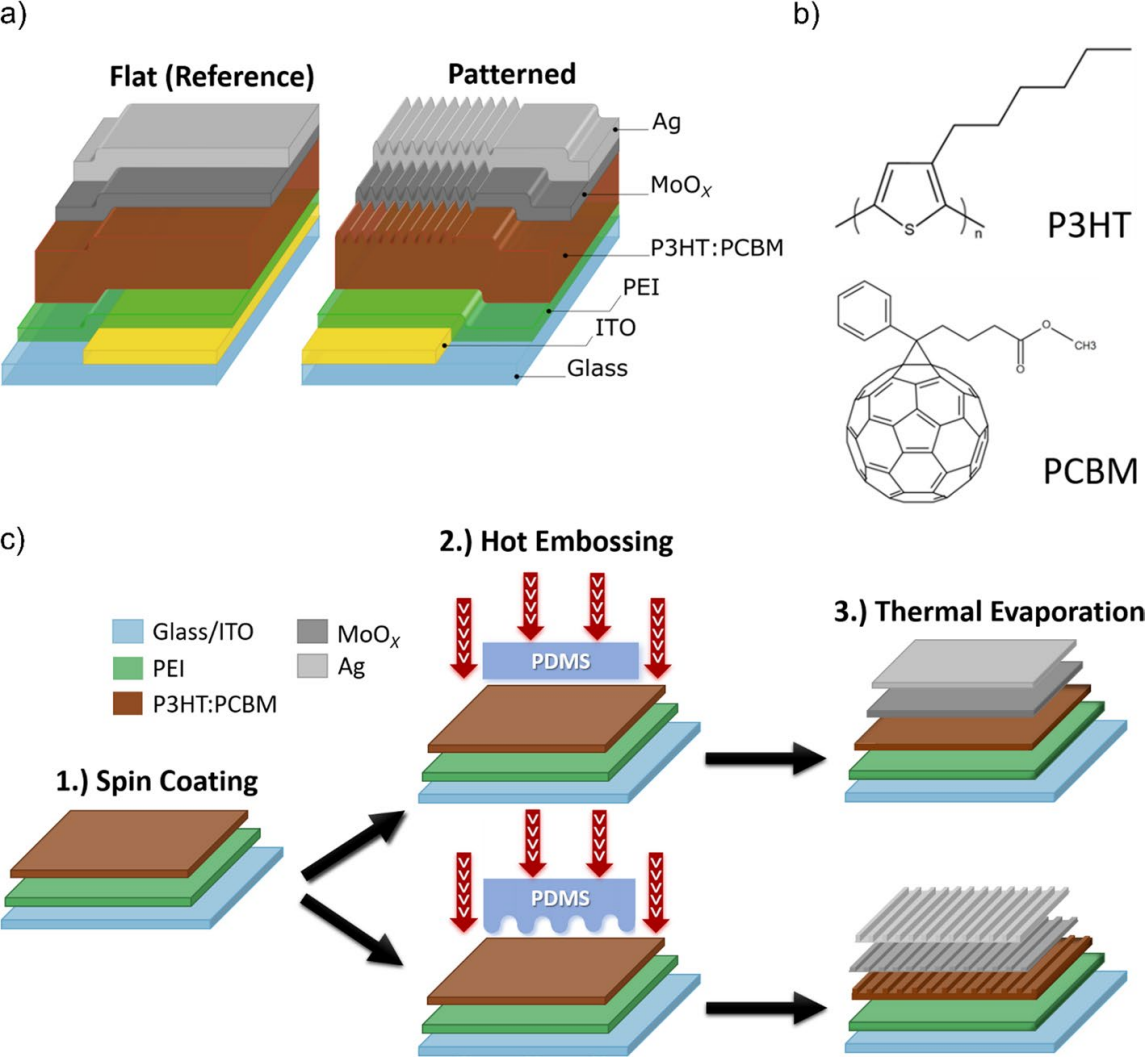
**a** Layer sequence of the investigated inverted bulk heterojunction solar cell devices. **b** Molecular structure of the P3HT polymer and the PCBM fullerene-derivate composing the photoactive blend. **c** Schematic illustration of the applied device preparation routine

For a thorough assessment regarding the impact of patterning the solar cell back electrode interface on the device performance, series of experiments were carried out with three different sets of devices. Respective device sets were prepared through an optimized protocol but differed by the structure superimposed over the metallic back electrode: set (A) featured a planar electrode, (B) was periodically patterned with a period (Λ) of 300 nm while set C) featured a Λ of 400 nm. Before conducting this study, great efforts were invested in establishing standardized, reproducible and comparable fabrication processes for realizing the patterned and reference cells before fabricating the devices shown here. All results presented in the following originate from a final series of device sets, while the characteristic of each device set was obtained by averaging the results obtained from six individual solar cells. Investigated device stacks are illustrated in Fig. 1a and consist of the layer sequence glass/ITO/PEI/P3HT:PCBM/MoO_*X*_/Ag, with ITO/PEI and MoO_*X*_/Ag forming the device cathode and anode, respectively (i.e., inverted solar cell device architecture). Examined devices utilize the well-characterized P3HT:PCBM photoactive blend with regioregular poly(3-hexylthiophene-2,5-diyl) (P3HT) as the electron donor and [6,6]-phenyl-C61-butyric acid methyl ester (PCBM) as the electron acceptor material (see Fig. 1b) [5, 48–52]. For P3HT:PCBM solar cells, (thermal) annealing the active layer is a mandatory fabrication step to improve the nano-morphology of the active layer and thereby to optimize the obtained device performance [53, 54]. In our investigations, thermal annealing was also used for imprinting the periodic 2D grating profile into the active layer top surface through a PDMS mold as illustrated by the process flow chart in Fig. 1c. Notably, the surface of the PDMS molds used in this study only partially bears the grating profile, while the remaining surface areas are flat. Employing such stamp design ensures that the characteristics of planar and patterned solar cells is attributed to the surface geometry of the back electrode interface and not to deviations in the preparation process (e.g., the effect of the PDMS stamp during thermal annealing) [55].

A. The photoactive layer thickness-performance dependence of planar and patterned solar cells

**Fig. 2:**
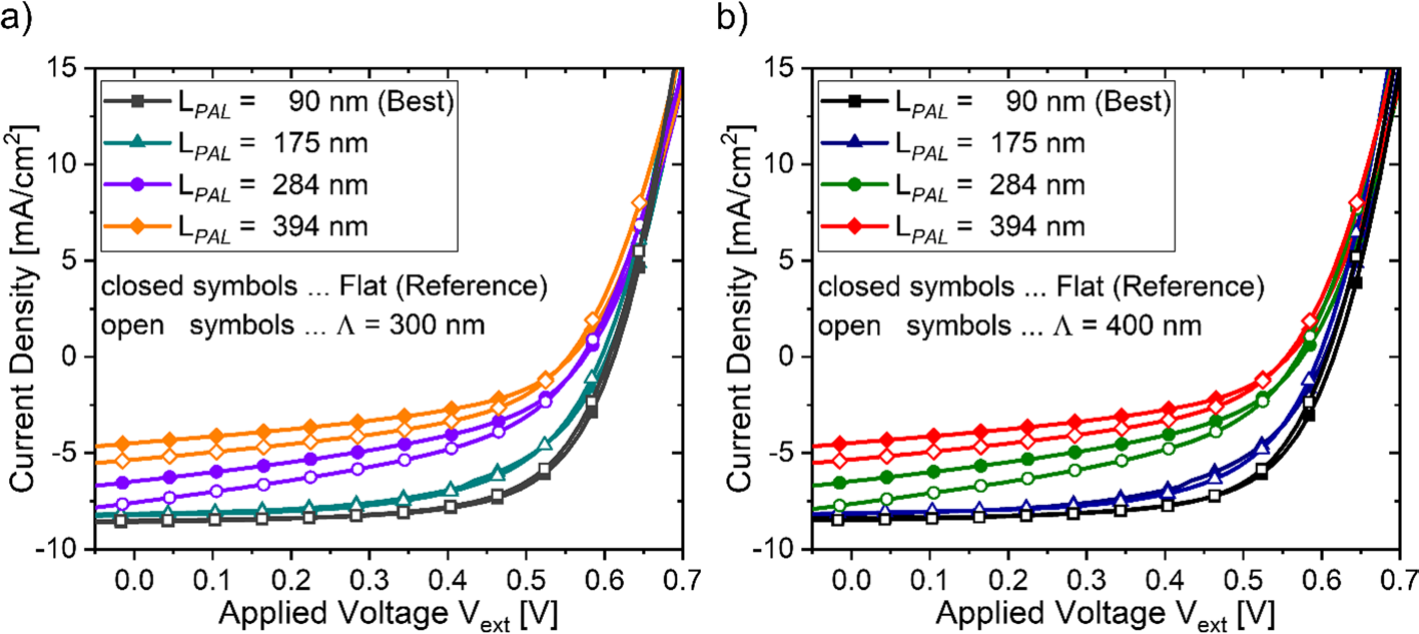
Measured JV characteristics of planar (closed symbols) and patterned (open symbols) solar cells in dependence of the gradually increased active layer thickness (L_*PAL*_). JV curves obtained from patterned solar cells bearing a pattern periodicity (Λ) of 300 nm and 400 nm are presented in (**a**) and (**b**), respectively

For both planar and patterned solar cells, the thickness of the photoactive layer (L_*PAL*_) was varied in the range between 90 and 400 nm to examine the device performance in dependence of L_*PAL*_. For each of the three investigated device sets (i.e., planar, patterned with a $\Lambda$ of 300 nm and 400 nm, respectively), we obtained the JV characteristic from six individual solar cells. The JV characteristics of the individual devices were used to evaluate the solar cell performance indicators (${V}_{OC}$, ${J}_{SC}$ and $FF$) and the power conversion efficiency (PCE) [56]. Fig. 2 presents the JV curve that was obtained by averaging the JV curves derived from the six individual solar cells devices and for a L_*PAL*_ of 90 ± 10 nm, 175 ± 12 nm, 284 ± 11 nm and 394 ± 16 nm, respectively. The averaged overall device performances and performance indicators of each device set are summarized in Table 1.

**Table 1:** Summary of the solar cell power conversion efficiency (PCE) and the corresponding individual performance indicators (${V}_{OC}$, open circuit voltage; ${J}_{SC}$, short circuit current density; $FF$, fill factor) of planar (i.e., flat) and patterned solar cells, derived for the respective active layer thicknesses (L_*PAL*_) used at our investigations

L_*PAL*_ [nm]	*V*_OC_ [V]			*J*_SC_ [mA/cm^2^]			FF []			PCE [%]		
	Flat	300	400	Flat	300	400	Flat	300	400	Flat	300	400
90 ± 10	0.61 (0.01)	0.61 (0.01)	0.61 (0.01)	8.6 (0.1)	8.5 (0.1)	8.5 (0.1)	0.65 (0.01)	0.65 (0.01)	0.64 (0.01)	3.4 (0.1)	3.3 (0.1)	3.3 (0.1)
175 ± 12	0.60 (0.01)	0.60 (0.01)	0.60 (0.01)	8.2 (0.1)	8.2 (0.1)	8.1 (0.1)	0.58 (0.01)	0.59 (0.01)	0.61 (0.01)	2.9 (0.1)	2.9 (0.1)	3.0 (0.1)
284 ± 11	0.58 (0.01)	0.57 (0.01)	0.57 (0.01)	6.5 (0.2)	7.6 (0.4)	7.6 (0.5)	0.44 (0.01)	0.45 (0.01)	0.46 (0.01)	1.6 (0.1)	1.9 (0.1)	1.9 (0.2)
394 ± 16	0.56 (0.01)	0.56 (0.01)	0.56 (0.01)	4.5 (0.2)	5.3 (0.3)	5.3 (0.3)	0.44 (0.01)	0.46 (0.01)	0.45 (0.01)	1.1 (0.1)	1.4 (0.1)	1.3 (0.1)

Presented results were obtained by avergaing the device performance and the performance indicators from six individual solar cell devices. The standard deviation is indicated in brackets below the averaged device performance parameters

The highest device performance was observed at a L_*PAL*_ of 90 nm, with a PCE of 3.4 ± 0.1% obtained from planar solar cells, while patterned devices showed for a very similar PCE of 3.3 ± 0.1%, regardless of the applied pattern periodicity. Gradually increasing the active layer thickness from 90 to 390 nm led to a dramatic decrease of the PCE, independently of the geometric surface profile (i.e., planar or patterned) of the solar cell back electrode interface. Lowering of the PCE when increasing L_*PAL*_ from a reference thickness of 90 nm is linked to (i) a small reduction of the open circuit voltage (${V}_{OC}$, ${\Delta V}_{OC}$ ~ 50 mV), (ii) a steep drop of the fill factor (FF) from ≥ 0.6 to < 0.45 for a L_*PAL*_ ≥ 284 nm and (iii) a continuous decrease of the short circuit current density (${J}_{SC}$). Interestingly, even though the thickness-related PCE decrease of planar and patterned solar cells follows a similar trend, the benefit of a patterned electrode interface becomes only apparent for a L_*PAL*_ ≥ 284 nm, manifesting in a relative PCE improvement of more than 15% due to an increased ${J}_{SC}$ as compared to a planar electrode interface. It is worth noting that applying a pattern periodicity of Λ = 300 nm or Λ = 400 nm does not seem to alter the device performance or the extracted photocurrent yields of the investigated patterned solar cells. With respect to planar devices, the higher ${J}_{SC}$ of patterned solar cells could be related to an optically induced photocurrent enhancement due to an increased device absorption, stemming from the excitation surface plasmon and dielectric waveguide modes. Yet, since the photocurrent enhancement of patterned solar cells is only observable for a L_*PAL*_ ≥ 284 nm, the performance improvement could be also related to an alternative mechanism. In previous reports, the performance decay when thickening the active layer, as observed at our investigated samples, was attributed to a decreased extraction efficiency of light induced photocarriers due to an increased non-geminate recombination mechanism [57–61]. Therefore, the higher ${J}_{SC}$ of patterned solar cells could be linked to a reduced charge carrier recombination and thus to an improved photocarrier extraction efficiency in presence of a patterned back electrode interface, compared to solar cell devices featuring a planar back electrode interface.

To gain further insights regarding the origin of the photocurrent enhancement in patterned OSCs and to clarify why applying different pattern periodicities does not affect the observed performance improvements, a twofold approach was applied to our investigations. First, the absorption characteristic of planar and Λ = 300 nm, 400 nm patterned solar cells is numerically modelled through a finite-difference time-domain (FDTD) approach for a L_*PAL*_ varied in the range between 90 and 400 nm. Obtained simulation outcomes are supplemented by an evaluation of the spectral dependent external quantum efficiency (EQE), obtained from fabricated planar and patterned solar cells at short circuit conditions (i.e., externally applied bias equals to zero). Second, to acquire a profound understanding of the photocarrier extraction characteristics of fabricated solar cells we compare the voltage-dependent experimental photocurrent characteristic $\left({J}_{ph}\left({V}_{\mathrm{eff}}\right)\right)$ with respect to (i) the thickness of the active layer and (ii) the geometric surface profile of the back electrode interface (i.e., planar vs. Λ = 300 nm, 400 nm patterned). Our findings on the simulated absorption and the experimental EQE characteristics are discussed in the following section, while the experimental ${J}_{ph}\left({V}_{eff}\right)$ characteristic of planar and patterned solar cells are presented in section 0.

B. Simulated absorption spectra and experimental external quantum characteristics

The optical characteristic of planar and patterned solar cells was numerically modelled by a finite-difference time-domain (FDTD) approach for a device layer sequence comprised of ITO/P3HT:PCBM/MoO_*X*_/Ag (see Fig. S2). The device layer stack used in the simulations is very similar to the layer sequence of fabricated solar cell devices (i.e., ITO/PEI/P3HT:PCBM/MoO_*X*_/Ag), except from the PEI layer which was omitted in our modelling approach. The absence of the PEI layer is, however, not expected to significantly affect the derived optical spectra as the PEI layer thickness is rather small (< 3 nm) with respect to the thickness of the remaining solar cell layers [62]. Moreover, to address the polarization sensitivity of the surface profile used for patterning the solar cell back electrode (i.e., a linear sinusoidal grating profile) [63], optical simulations of planar and patterned solar cells were performed for incident light in TM- and TE-polarization, respectively. The optical characteristics presented in the following were obtained by averaging the simulation results from the respective (TM-,TE-) light polarization states.

**Fig. 3:**
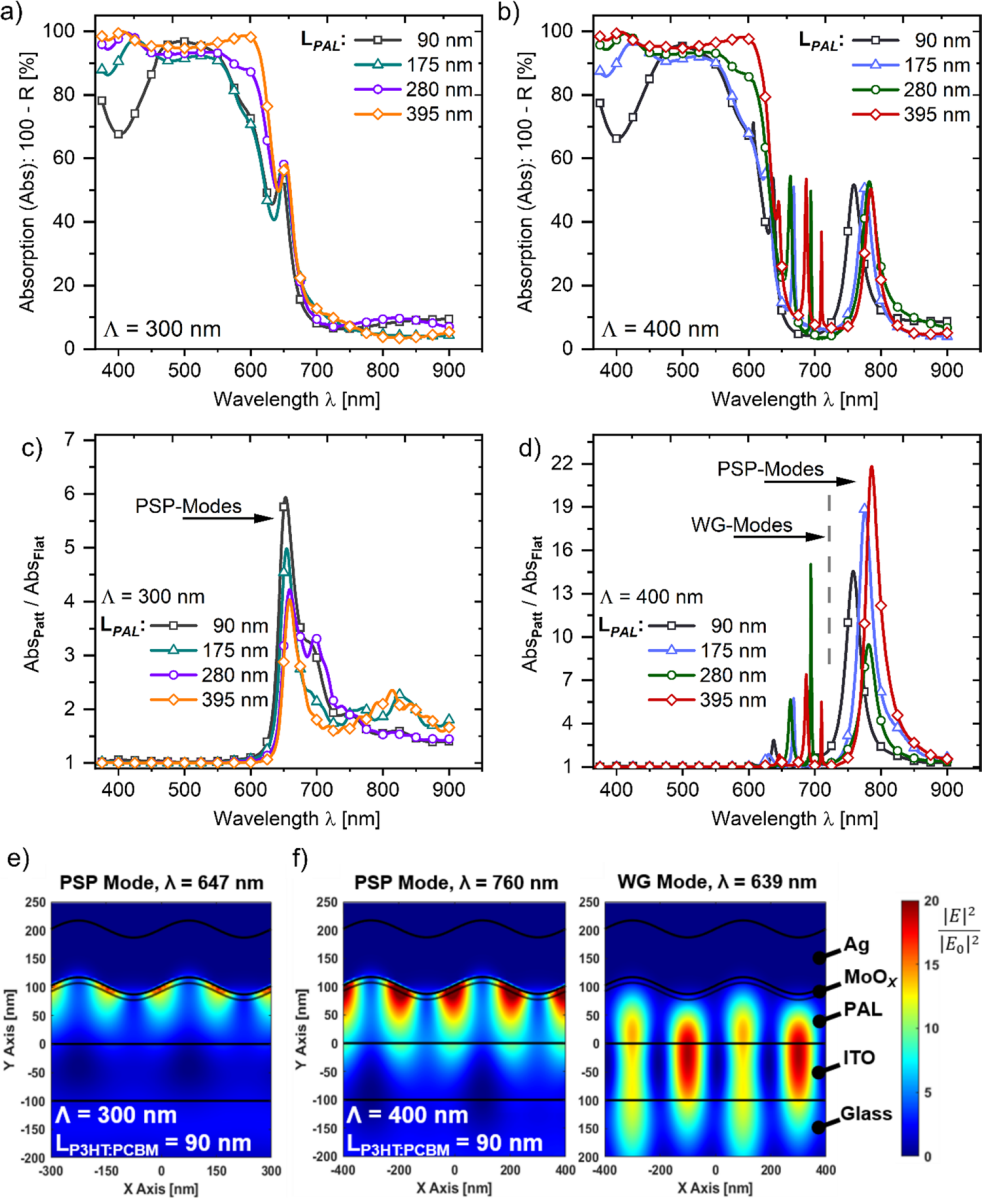
Simulated absorption characteristic obtained from a varied active layer thickness (L_*PAL*_) of patterned solar cells featuring a pattern periodicity (Λ) of (**a**) 300 nm and (**b**) 400 nm, respectively. Relative absorption enhancements, defined as the ratio of the absorption characteristics observed for planar (i.e., flat) and patterned solar cells and for an applied pattern periodicity of (**c**) 300 nm and (**d**) 400 nm. Spatial distribution of the optical near-field enhancements, particularly obtained for a L_*PAL*_ of 90 nm for light coupling to a (TM-polarized) propagating surface plasmon (PSP) or to a (TE-polarized) dielectric waveguide (WG) mode of (**e**) Λ = 300 nm or (**f**) Λ = 400 nm patterned solar cells

Comparing the simulated absorption characteristic, particularly obtained for an active layer thickness of 90, 175, 280 and 395 nm of planar (see Fig. S3a) and Λ = 300 nm, 400 nm patterned solar cells (see Fig. 3a, b, respectively) shows that increasing L_*PAL*_ from a reference thickness of 90 nm significantly improves the light absorption at wavelengths < 640 nm. The absorption improvement when increasing the thickness of the active layer is observed for both planar and patterned solar cells, and seems to be rather linked to the active layer thickness but not to the geometric surface profile of the back electrode interface. Yet, we find the absorption characteristic of patterned solar cells (Fig. 3a, b) modified by the introduction of additional absorption peaks at wavelengths > 625 nm compared to the light absorption characteristics of planar solar cells (Fig. S3a). Patterning the back electrode interface with a periodicity of 300 nm induces a more than fivefold improvement of the absorption at the center peak wavelength (${\lambda }_{CP}$) as highlighted in Figure 3c. Applying a pattern periodicity of 400 nm results in a more than 20-fold absorption improvement as suggested by our simulation findings (see Fig. 3d). Interestingly, thickening the active layer has a different impact on the absorption enhancements with respect to the applied pattern periodicity. For Λ = 300 nm patterned solar cells, increasing L_*PAL*_ from our reference thickness of 90 nm induces a lowering of the observed absorption enhancements (see Fig. 3c). This characteristic was, however, not observed at Λ = 400 nm patterned solar cells, where increasing L_*PAL*_ (i) induces a spectral red-shift of ${\lambda }_{CP}$ (not observed at Λ = 300 nm solar cells) and (ii) results in the introduction of additional absorption speaks at wavelengths > 625 nm (see Fig. 3d).

Based on the spatial distribution of the simulated optical near-field enhancements, defined as the ratio of the square electric field amplitude inside the structure and of the incident plane wave (${\left|E\right|}^{2}/{\left|{E}_{0}\right|}^{2}$), we can safely link the absorption improvements of patterned solar cells to the resonant coupling of light to (i) propagating surface plasmon (PSP) modes (Λ = 300 nm, 400 nm) and (ii) dielectric waveguide (WG) modes (Λ = 400 nm only). Compared to the spatial near-field intensity enhancement distribution of planar solar cells (see Fig. S3), excitation of a PSP mode induces a strong spatial confinement of the incident optical fields in close vicinity to the corrugated active layer/back electrode interface as depicted in Fig. 3e and f for an applied pattern periodicity of 300 nm and 400 nm, respectively. The spectral position, where the resonant coupling of normally incident light to a PSP mode occurs can be predicted using the phase matching relation $Re\left\{\left.{k}_{psp}\right\}\right.= m 2\pi /\Lambda$, where Λ is the pattern periodicity of the periodically corrugated dielectric/metal interface, while m is the diffraction order [13]. Consequently, increasing the pattern periodicity induces a spectral red-shift of the PSP modes as manifested by the shift of the absorption enhancements from a wavelength of 647 nm to wavelengths > 740 nm when applying a pattern periodicity of 300 nm and 400 nm, respectively (see Fig. 3c, d). Applying these periodicities, therefore, allows us to evaluate the impact of coupling to PSP modes on the light absorption and, correspondingly, on the photocurrent generation, where the P3HT:PCBM photoactive layer blend shows for a strong (*λ* < 650 nm) or a weak (*λ* > 700 nm) absorption. The wavelength dependent light absorption characteristic of the photoactive layer could also explain the weaker absorption enhancements of Λ = 300 nm patterned solar cells (fivefold) to that of Λ = 400 nm patterned solar cells (20-fold). The higher extinction coefficient of the P3HT:PCBM blend at wavelengths < 650 nm (see Fig. S2) induces a stronger damping of the PSP mode that renders the excited surface plasmon modes less effective at such wavelength ranges [64].

Aside from the coupling to PSP modes, patterning the active layer/back electrode interface with a periodicity of 400 nm additionally allows for the excitation of (multiple) dielectric waveguide modes, according to our simulations. With respect to the optical near-field enhancements simulated for the excitation of PSP modes, WG modes show for more delocalized near field enhancements, overlapping with device layers adjacent to the photoactive layer and in particular with the high refractive index ITO electrode (see Fig. 3f). Moreover, when increasing the active layer thickness, the number of WG modes increases (see Fig. S4, particularly obtained for a L_*PAL*_ of 280 nm), leading to a series of sharp absorption peaks at wavelengths below the wavelength associated with the resonant coupling to PSP modes. For the sake of completeness, it is further noted that due to the polarization sensitivity of the linear grating profile used for patterning the back electrode interface, coupling to dielectric WG modes is observed for both TM- and TE-polarized light, whereas excitation of a PSP mode is observed for light in TM-polarization only. To evaluate the overall absorption enhancement when patterning the active layer/back electrode interface, we integrated the absorption characteristics obtained from patterned solar cells over the wavelength range used in our investigations (i.e., *λ* = 350–900 nm) and compared the results to the integrated absorption characteristic of planar solar cells. Applying a pattern periodicity of 300 nm/400 nm results, with respect to the utilized active layer thickness, in a relative overall absorption enhancement by + 14.4%/ + 12.7% (L_*PAL*_ = 90 nm), + 10.8%/ + 11.3% (L_*PAL*_ = 175 nm), + 9.6%/ + 10.9% (L_*PAL*_ = 280 nm) and + 7.5%/ + 9.3% (L_*PAL*_ = 390 nm). Yet, the absorption enhancements when patterning the active layer/back electrode interface do not necessarily translate in an equivalent photocurrent improvement. As shown by our examination of the spatial near-field intensity enhancements distribution, the intensities of the incident optical fields are not only enhanced within the active layer region but also within the metal back electrode or the ITO front electrode when exciting PSP- or WG-modes, respectively (see Figs. 3e, f and S4). Enhancing the optical E-field intensities and thereby the absorbed optical power in the respective electrode layers promotes, however, only the parasitic absorption characteristic of the investigated solar cell layout but does not contribute in a higher photocurrent generation of the device [65–67].

**Fig. 4:**
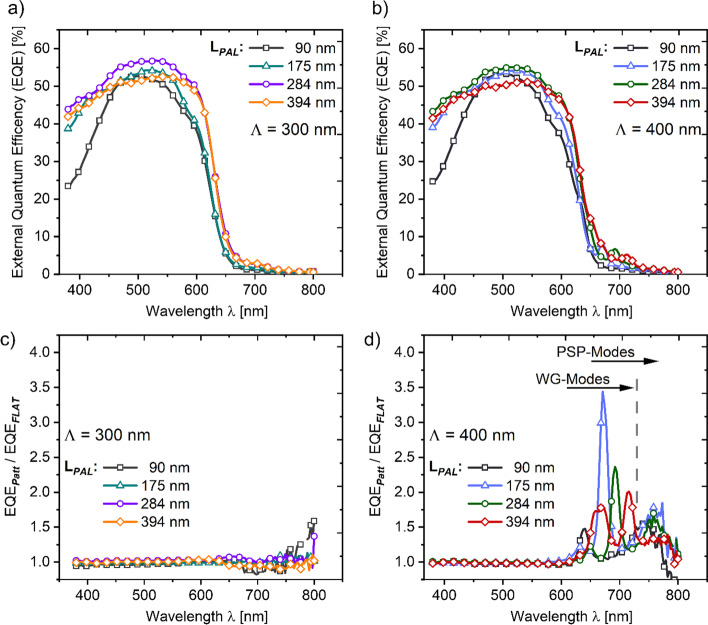
External quantum efficiency characteristic of fabricated patterned solar cell devices featuring a pattern periodicity (Λ) of (**a**) 300 nm and (**b**) 400 nm, respectively. Relative EQE enhancements (i.e., the ratio of the EQE characteristics observed for patterned and planar (i.e., flat) solar cells) of (**c**) Λ = 300 nm and (**d**) Λ = 400 nm patterned solar cells

To gain a better insight regarding the photocurrent generation in dependence of the wavelength of normally incident light, we additionally evaluated the external quantum efficiency (EQE) characteristic of fabricated planar (see Fig. S3b) and Λ = 300, 400 nm patterned solar cells (see Fig. 4a and b). In agreement with our previously presented simulation results, we find the EQE characteristics of both planar and patterned solar cells enhanced at spectral ranges < 640 nm when increasing the active layer thickness from a reference thickness of 90 nm [60, 68]. Compared to the EQE of planar solar cells, patterning the solar cell back electrode with a periodicity of 400 nm improved the EQE characteristic at wavelength ranges > 640 nm (see Fig. 4d). The EQE improvements of Λ = 400 nm patterned solar cells at these spectral ranges originate from the coupling of incident light to PSP- and WG-modes that improve the photocurrent generation through an increased light absorption in the P3HT:PCBM layer as proposed by our simulations. In addition, increasing the photoactive layer thickness of fabricated Λ = 400 nm patterned solar cells also spectrally red-shifts the PSP- and WG-mode-related EQE enhancements, a characteristic we find in a good agreement with our simulation outcomes. Yet, the surface plasmon- and waveguide-induced EQE enhancements of fabricated Λ = 400 nm patterned solar cells are significantly lower compared to the absorption enhancements observed at our simulation approach. Lowering of the PSP- and WG-mode-related EQE enhancements of Λ = 400 nm patterned solar cells may on one hand originate from an increased parasitic absorption characteristic of fabricated solar cells when exciting respective optical modes. On the other hand, the lowered EQE enhancements might also indicate for a reduced coupling efficiency of incident light to the respective PSP- and WG-modes at the fabricated devices, probably caused by an imperfect quality of the pattern imprint (see Fig. S1). However, and most surprisingly, the EQE characteristics of Λ = 300 nm patterned solar cells is almost identical to the EQE characteristics of planar solar cells and thus does not evidence for an (optically) enhanced photocurrent generation due to the excitation of a PSP- mode as suggested by our simulations. The absence of a PSP-mode-related EQE enhancement at Λ = 300 nm patterned solar cells might be linked, again, to an imperfect imprint quality of the pattern structure and/or to an even higher optical mode damping of the P3HT:PCBM blend in fabricated solar cells at spectral ranges < 650 nm.

Since in our investigations, the EQE characteristic was acquired for an externally applied bias of zero (i.e., short circuit conditions), integrating the obtained EQE spectra over the wavelength range used in our investigations (*λ* = 375–800 nm), while assuming a light intensity of unity, allows for a rough estimation of the short circuit current density (${J}_{SC, EQE}$) [68]. Comparing the ${J}_{SC, EQE}$ of Λ = 400 nm patterned solar cells with the ${J}_{SC, EQE}$ of planar solar cells for a similar active layer thickness indicates a quite meager relative photocurrent improvement of less than 1% when patterning the back electrode. We can, therefore, safely assume that the higher ${J}_{SC}$ of patterned solar cells, as listed in Table 1, does not originate from an optically induced enhancement but is related to an alternative mechanism. Moreover, the spectral broadening of the EQE characteristic when increasing the active layer thickness of both planar and (Λ = 300 nm, 400 nm) patterned solar cells does not follow the characteristic of the ${J}_{SC}$ with respect to the active layer thickness (i.e., lowering of ${J}_{SC}$ when increasing L_*PAL*_). The divergence of these two characteristics is possibly linked to the different irradiance (Φ) levels used at the derivation of the ${J}_{SC}$ performance indicator (Φ = 100 mW/cm^2^) and the EQE spectra (Φ <  < 100 mW/cm^2^). The higher photocurrents of patterned solar cells are thus possibly linked to an irradiance dependent mechanism, such as non-geminate charge recombination mechanisms [69]. As non-geminate recombination detrimentally affects the photocarrier extraction efficiency at low and intermediary bias ranges [70], we examine in the following the experimental voltage dependent photocarrier extraction characteristic of planar and patterned solar cells at irradiance levels that were gradually varied in the range between 1 and 100 mW/cm^2^.

C. Effective voltage dependent photocurrent characteristics

Evaluating the external quantum efficiency of fabricated planar and Λ = 300, 400 nm patterned solar cells unambiguously demonstrated that the higher short circuit current densities (${J}_{SC}$) of patterned solar cells is not related to an optically enhanced photocurrent generation. We, therefore, turn the focus of our discussions on the electrical characteristic of fabricated solar cells by examining the effective voltage dependent photocurrent characteristic (${J}_{ph}\left({V}_{eff}\right)$) based on the photocarrier extraction model that was initially developed by Goodman and Rose [70]. In this approach, the experimental photocurrent is defined as ${J}_{ph}= {J}_{light}- {J}_{dark}$ with ${J}_{light}$ and ${J}_{dark}$ as the electric current observed under illumination and dark conditions, respectively. The effective voltage driving the photocarriers for collection to the solar cell electrode is given as ${V}_{eff}= {V}_{0}- {V}_{ext}$ with ${V}_{ext}$ as the externally applied voltage and ${V}_{0}$ as the bias level, where ${J}_{ph}$ is equal to zero. Plotting ${J}_{ph}$ vs. ${V}_{eff}$ on a double-logarithmic scale allows to investigate the power law dependence of the voltage dependent photocurrent (${J}_{ph}\propto {V}_{eff}^{k}$) by examining the linear fitting coefficient ($k$) at different charge extraction regimes. [70–72].

**Fig. 5:**
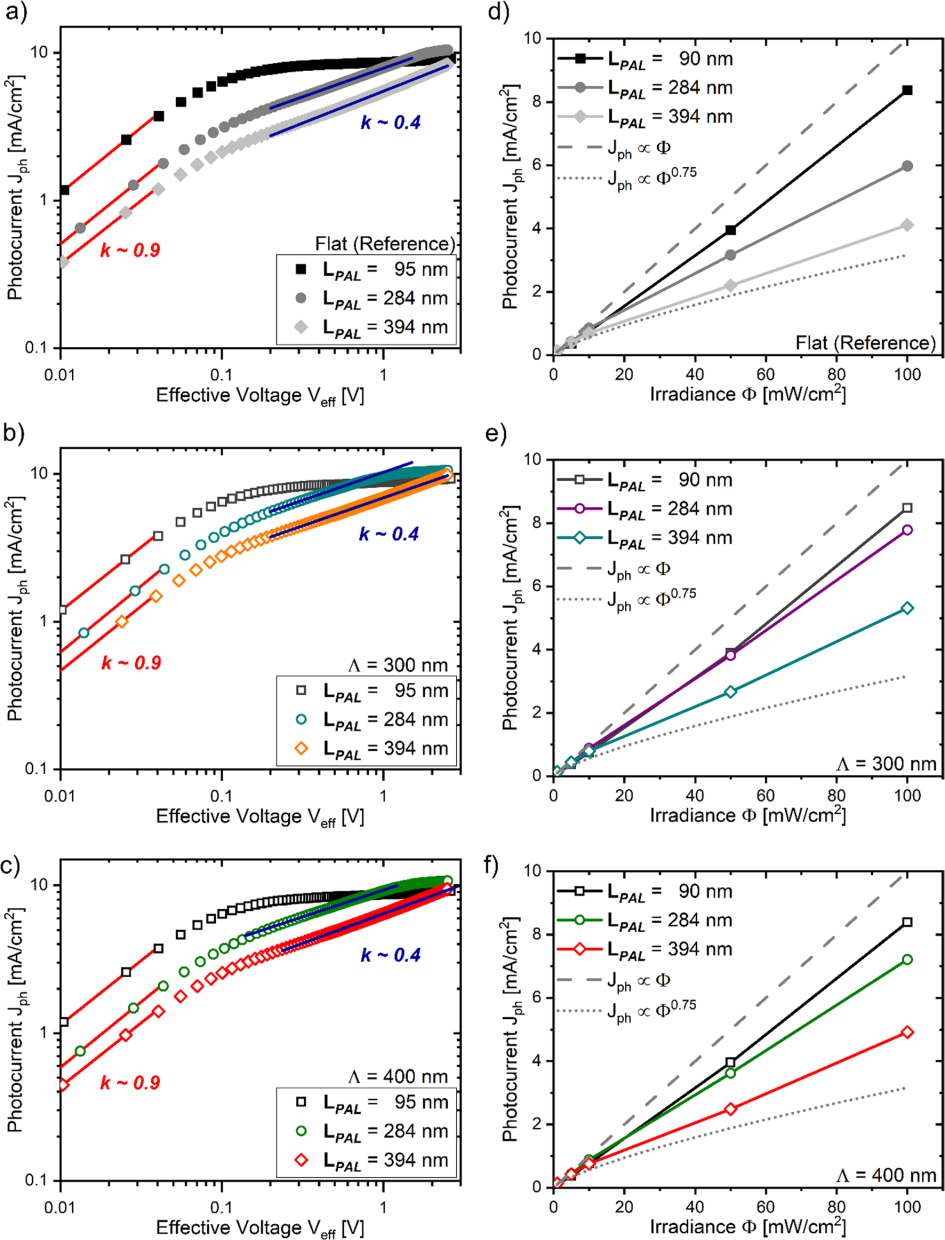
Voltage-dependent photocurrent characteristics on a double logarithmic scale, observed for a gradually increased active layer thicknesses (L_*PAL*_) of (**a**) planar, (**b**) Λ = 300 nm and (**c**) Λ = 400 nm patterned solar cells. Solid lines indicate the linear fitting functions used to evaluate the power law coefficient $k$. Irradiance dependent photocurrent characteristics obtained from (**d**) planar, (**e**) Λ = 300 nm and (**f**) Λ = 400 nm patterned solar cells. Photocurrents strictly scaling to light intensities in a linear fashion (${J}_{ph}\propto \Phi$) or by a three-quarter power coefficient (${J}_{ph}\propto {\Phi }^{0.75}$) are highlighted by dashed and dotted curves, respectively

The ${J}_{ph}\left({V}_{eff}\right)$ characteristics of planar and patterned solar cells that was particularly obtained at an irradiance of 100 mW/cm^2^ are presented in Fig. 5a–c. Independently of the active layer thickness (L_*PAL*_), a linear fitting coefficient close to unity ($k$ = 0.9) is observed for a ${V}_{eff}$ < 0.1 V for both planar and patterned solar cells, indicating for an ohmic charge extraction at such low ${V}_{eff}$ levels (i.e., ${J}_{ph}\propto {V}_{eff}$). At higher ${V}_{eff}$ levels, however, the charge extraction regimes differ significantly with respect to the active layer thickness. For planar and patterned solar cells featuring a L_*PAL*_ ≤ 175 nm, the photocurrent shows for a saturated characteristic at ${V}_{eff}$ > 0.2 V ($k$ = 0). Hence, the ${J}_{ph}\left({V}_{eff}\right)$ characteristics acquired for fabricated solar cells with a ${L}_{PAL}$ ≤ 175 nm evidence for a photocarrier extraction that is unaffected by a (non-geminate) recombination mechanism. In contrast, a power coefficient of 0.4 is derived at intermediary ${V}_{eff}$ levels (i.e., 0.2 < ${V}_{eff}$  < 1 V), while saturation of the photocurrent is shifted to a ${V}_{eff}$ > 1 V at solar cells with a L_*PAL*_ ≥ 284 nm. The fitting coefficient obtained at intermediary ${V}_{eff}$ levels suggests for a square-root dependence of ${J}_{ph}$ on ${V}_{eff}$ (i.e., ${J}_{ph}\propto \sqrt{{V}_{eff}}$) and thus for a photocarrier extraction impaired by non-geminate recombination or by the introduction of space charges [70]. The occurrence of a square-root photocurrent regime further explains the previously observed performance decay of planar and patterned solar cells when using a L_*PAL*_ ≥ 284 nm (see Table 1). Here, the performance decay when increasing the active layer thickness was caused by a lowering of the fill factor and short circuit current densities, since both performance indicators are derived at ${V}_{eff}$ levels, where ${J}_{ph}\left({V}_{eff}\right)$ indicates for a square-root regime.

Assigning the square root photocurrent dependence to a photocarrier extraction limited by non-geminate recombination or space charges requires the acquisition of the ${J}_{ph}\left({V}_{eff}\right)$ characteristic at different irradiance levels (Φ) of the incident light [70]. Accordingly, we obtained the voltage dependent photocurrent characteristic of our solar cells for irradiance levels that were gradually varied through a set of neutral density filters in the range between 1 and 100 mW/cm^2^ (see Figs. S6 and S7). Independently of *L*_PAL_, the ${J}_{ph}\left({V}_{eff}\right)$ characteristic obtained at Φ < 10 mW/cm^2^ from both planar and patterned solar cells switches between an ohmic at low ${V}_{eff}$ and a saturated photocarrier extraction regime when reaching higher ${V}_{eff}$ levels. Applying an Φ > 10 Mw/cm^2^ does not modify the ${J}_{ph}\left({V}_{eff}\right)$ characteristic of planar and patterned solar cells with a L_*PAL*_ ≤ 175 nm, whereas a square-root photocurrent regime is observed at intermediary ${V}_{eff}$ levels for solar cells with a L_*PAL*_ ≥ 284 nm. Extracting the photocurrent yields at a ${V}_{eff}$ level equivalent to short circuit condition (i.e., ${V}_{eff}$ ~ 0.5–0.7 V, depending on Φ) and plotting ${J}_{ph}$ vs. Φ reveals a linear dependence of ${J}_{ph}$ on Φ for a L_*PAL*_ = 90 nm (see Fig. 5d–f). In contrast, for solar cells featuring a L_*PAL*_ ≥ 284 nm, the ${J}_{ph}$ obtained at short circuit conditions scales non-linearly with Φ, gradually approaching a three-quarter dependence of ${J}_{ph}$ on Φ for irradiance levels > 10 mW/cm^2^. The occurrence of a square root photocurrent regime together with the three-quarter dependence of ${J}_{ph}$ on Φ clearly evidences the introduction of space charges at planar and patterned solar cells when using a L_*PAL*_ ≥ 284 nm.

**Fig. 6:**
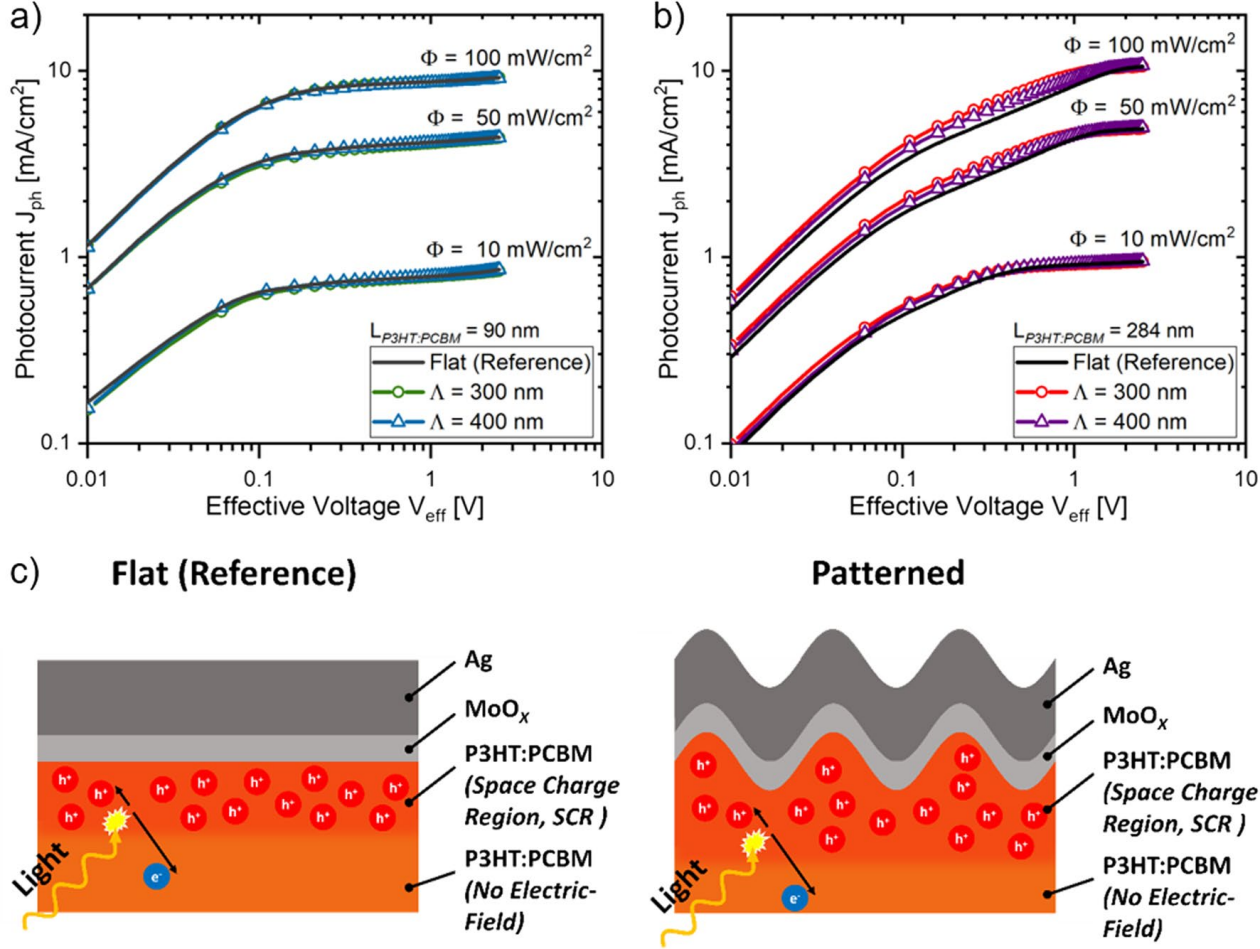
${J}_{ph}\left({V}_{eff}\right)$ characteristics of planar and Λ = 300, 400 patterned solar cells observed at different light intensity (Φ) levels and at an active layer thickness (${L}_{PAL}$) of **a** 90 nm and **b** 284 nm, respectively. Graphical illustration of the space charge region size of planar (flat) and patterned back electrode interfaces (**c**)

Figure 6 presents a comparison of the experimental ${J}_{ph}\left({V}_{eff}\right)$ characteristic observed at a Φ of 10, 50 and 100 mW/cm^2^ for planar and patterned solar cells, bearing an active layer thickness, where charge extraction was unaffected through charge recombination (L_*PAL*_ = 90 nm) and for a space charge-induced recombination mechanism (L_*PAL*_ = 284 nm). For both applied pattern periodicities (i.e., Λ = 300 nm, 400 nm), corrugating the active layer/back electrode interface improves the extracted photocurrents yields at the ohmic and the space charge limited (SCL) extraction regime when using a L_*PAL*_ ≥ 284 nm compared to planar solar cells. In contrast, similar photocurrent yields are extracted from planar and patterned devices, where the extraction of light-induced charge carriers is not affected by space charges. For P3HT:PCBM solar cells in particular, the occurrence of space charges was recently linked to an imbalanced charge carrier mobility in P3HT and PCBM, resulting in the accumulation of the slower photocarrier species (i.e., holes) at the device’s anode [73]. The accumulating holes induce a split-up of the (presumed) homogeneously distributed charge collection fields into a space charge region (SCR) containing the whole charge collection field and into a quasi-neutral region devoid of any electric fields. As a consequence, only photocarriers generated within the SCR are extracted at the respective device electrodes, while photocarriers generated in the quasi-neutral region are not subject of an electric charge collection field and eventually recombine. For solar cells exhibiting a SCL photocarrier extraction, the magnitude of the extracted photocurrent yields depend on the size of the space charge region; increasing the size of the SCR results in an improved photocurrent yield [70]. Accordingly, our findings suggest that the higher photocurrent yields of Λ = 300 nm, 400 nm patterned solar cells, particularly observed in the SCL extraction regime, is linked to an increased size of the space charge region. The increase of the space charge region size of the patterned devices is possibly caused by the different surface profile of the solar cell back electrode interface compared to planar solar cells as illustrated in Fig. 6 c. Interestingly, the excitation of a surface plasmon mode at the back electrode interface, evidenced through an increased photocurrent generation at the EQE characteristic of Λ = 400 nm patterned solar cells (see Fig. 4b), did not result in a break-up of the space charge regime in contrast to previous findings [38]. Overall, presented results strongly suggest that patterning the back electrode interface with a Λ = 300 nm, 400 nm at inverted P3HT:PCBM solar cells improves the extracted photocurrent yields by increasing the active layer regions covered by the electric charge collection fields (i.e., the space charge region) in the SCL extraction regime. The observed photocurrent improvements when patterning the solar cell back electrode interface, however, could not be linked to an optically induced photocurrent enhancement or a spatial redistribution of the photocurrent generation profile, caused by the excitation of surface plasmon or dielectric waveguide modes.

## Conclusion

The impact of a planar (i.e., flat) and a periodically patterned active layer/back electrode interface on the generation and the extraction of light induced photocurrents has been examined for inverted ITO/PEI/P3HT:PCBM/MoO_*X*_/Ag solar cells, where the photoactive layer thickness (L_*PAL*_) was gradually varied in the range between 90 and 400 nm. Our investigations focused on a pattern structure that consisted of a (linear) 2D sinusoidal grating profile and two different pattern periodicities (Λ = 300 nm, 400 nm) were used for patterning the solar cell back electrode interface. Numerically modelling the light absorption characteristic of planar and patterned solar cells through a finite-difference time-domain (FDTD) approach demonstrated for an increased device absorption in presence of a patterned electrode interface, compared to the light absorption characteristic of planar solar cells. The higher light absorption of patterned solar cells originates from the excitation of surface plasmons (Λ = 300 nm, 400 nm) and dielectric waveguide modes (Λ = 400 nm only). Simulation results were further validated by examining the experimental external quantum efficiency (EQE) characteristic of fabricated planar and patterned solar cells. The EQE characteristic of Λ = 400 nm patterned solar cells evidenced an enhanced photocurrent generation that was caused by the resonant coupling of incident light to surface plasmon and waveguide modes, whereas Λ = 300 nm patterned solar cells did not show for an improved photocurrent generation compared to planar devices. However, our evaluation of the EQE spectra could not explain the higher short circuit densities (${J}_{SC}$) and, consequently, the higher power conversion efficiencies (PCE) of Λ = 300 nm, 400 nm patterned devices with respect to the PCE and ${J}_{SC}$ of planar devices when using a L_*PAL*_ ≥ 284 nm. To examine the different charge extraction regime of fabricated solar cells we analyzed the voltage dependent experimental photocurrent characteristic ${J}_{ph}\left({V}_{eff}\right)$ at irradiance levels varied in the range between 1 and 100 mW/cm^2^. Comparing the ${J}_{ph}\left({V}_{eff}\right)$ characteristic of planar and patterned solar cells revealed that patterned devices show for higher photocurrent yields in the space charge limited (SCL) extraction regime, independently of the applied pattern periodicity. Corrugating the surface profile of the solar cell active layer/back electrode interface apparently increases the size of the space charge region, containing the entire charge collection fields in the SCL regime, with respect to the space charge region size of solar cells bearing a planar active layer/back electrode interface. We believe that our presented findings do not only improve the understanding how patterning the active layer/electrode interface affects the photocarrier extraction at the solar cell SCL extraction regime but may also pave the way for evaluating the charge mobility of organic semiconductors through the space charge limited current method in diode configuration.

## Supplementary Information

Below is the link to the electronic supplementary material.

Supplementary file1 (DOCX 1944 KB)

## Data Availability

Data sets acquired and/or analyzed during the presented study are available from the corresponding author on reasonable request.

## References

[1] Riede M, Spoltore D, Leo K (2021). Organic solar cells—the path to commercial success. Adv Energy Mater..

[2] Xue R, Zhang J, Li Y, Li Y (2018). Organic solar cell materials toward commercialization. Small.

[3] Yang F, Huang Y, Li Y, Li Y (2021). Large-area flexible organic solar cells. npj Flex Electron..

[4] Hong L, Yao H, Cui Y, Ge Z, Hou J (2020). Recent advances in high-efficiency organic solar cells fabricated by eco-compatible solvents at relatively large-area scale. APL Mater..

[5] Ghosekar IC, Patil GC (2021). Review on performance analysis of P3HT:PCBM-based bulk heterojunction organic solar cells. Semicond Sci Technol..

[6] Sadoogi N, Rostami A, Faridpak B, Farrokhifar M (2021). Performance analysis of organic solar cells: opto-electrical modeling and simulation. Eng Sci Technol Int J..

[7] Ajayan J, Nirmal D, Mohankumar P, Saravanan M, Jagadesh M, Arivazhagan L (2020). A review of photovoltaic performance of organic/inorganic solar cells for future renewable and sustainable energy technologies. Superlattices Microstruct..

[8] Zhang Y, Wu B, He Y (2022). Layer-by-layer processed binary all-polymer solar cells with efficiency over 16% enabled by finely optimized morphology. Nano Energy.

[9] Liu Q, Jiang Y, Jin K (2020). 18% Efficiency organic solar cells. Sci Bull..

[10] Amalathas AP, Alkaisi MM (2019). Nanostructures for light trapping in thin film solar cells. Micromachines..

[11] Guo CF, Sun T, Cao F, Liu Q, Ren Z (2014). Metallic nanostructures for light trapping in energy-harvesting devices. Light Sci Appl..

[12] Mokkapati S, Catchpole KR (2012). Nanophotonic light trapping in solar cells. J Appl Phys..

[13] Yu Z, Raman A, Fan S (2010). Fundamental limit of nanophotonic light trapping in solar cells. Proc Natl Acad Sci USA..

[14] Green M, Emery K, Hishikawa Y (2012). Solar cell efficiency tables (version 40). Ieee Trans Fuzzy Syst..

[15] Petoukhoff CE, Shen Z, Jain M, Chang A, O’Carroll DM (2015). Plasmonic electrodes for bulk-heterojunction organic photovoltaics: a review. J Photon. Energy..

[16] Atwater HA, Polman A (2010). Plasmonics for improved photovoltaic devices. Nat Mater..

[17] Ahn S, Rourke D, Park W (2016). Plasmonic nanostructures for organic photovoltaic devices. J. Opt. (United Kingdom)..

[18] Gan Q, Bartoli FJ, Kafafi ZH (2013). Plasmonic-enhanced organic photovoltaics: breaking the 10% efficiency barrier. Adv Mater..

[19] Shen H, Maes B (2011). Combined plasmonic gratings in organic solar cells. Opt Express..

[20] Sefunc MA, Okyay AK, Demir HV (2011). Plasmonic backcontact grating for P3HT:PCBM organic solar cells enabling strong optical absorption increased in all polarizations. Opt Express..

[21] Toan Dang P, Nguyen TK, Le KQ (2017). Revisited design optimization of metallic gratings for plasmonic light-trapping enhancement in thin organic solar cells. Opt Commun..

[22] Wen L, Sun F, Chen Q (2014). Cascading metallic gratings for broadband absorption enhancement in ultrathin plasmonic solar cells. Appl. Phys. Lett..

[23] Yousif B, Abo-Elsoud MEA, Marouf H (2019). Triangle grating for enhancement the efficiency in thin film photovoltaic solar cells. Opt. Quantum Electron..

[24] Chiu NF, Hou CH, Cheng CJ, Tsai FY (2013). Plasmonic circular nanostructure for enhanced light absorption in organic solar cells. Int. J. Photoenergy..

[25] Chriki R, Yanai A, Shappir J, Levy U (2013). Enhanced efficiency of thin film solar cells using a shifted dual grating plasmonic structure. Opt Express..

[26] Bi YG, Feng J, Chen Y (2015). Dual-periodic-corrugation-induced broadband light absorption enhancement in organic solar cells. Org Electron..

[27] Li X, Choy WCH, Ren X, Xin J, Lin P, Leung DCW (2013). Polarization-independent efficiency enhancement of organic solar cells by using 3-dimensional plasmonic electrode. Appl. Phys. Lett..

[28] Li K, Haque S, Martins A (2020). Light trapping in solar cells: simple design rules to maximize absorption. Optica..

[29] Wang C, Yu S, Chen W, Sun C (2013). Highly efficient light-trapping structure design inspired by natural evolution. Sci. Rep..

[30] Khan I, Keshmiri H, Kolb F, Dimopoulos T, List-Kratochvil EJWW, Dostalek J (2016). Multidiffractive broadband plasmonic absorber. Adv Opt Mater..

[31] Lin A, Phillips J (2008). Optimization of random diffraction gratings in thin-film solar cells using genetic algorithms. Sol. Energy Mater. Sol. Cells..

[32] Li XH, Sha WEII, Choy WCHH, Fung DDSS, Xie FX (2012). Efficient inverted polymer solar cells with directly patterned active layer and silver back grating. J. Phys. Chem C..

[33] Wang DH, Seifter J, Park JH, Choi DG, Heeger AJ (2012). Effi ciency increase in flexible bulk heterojunction solar cells with a nano-patterned indium zinc oxide anode. Adv Energy Mater..

[34] Hsu CM, Battaglia C, Pahud C (2012). High-efficiency amorphous silicon solar cell on a periodic nanocone back reflector. Adv. Energy Mater..

[35] Oh J, Yuan HC, Branz HM (2012). An 18.2%-efficient black-silicon solar cell achieved through control of carrier recombination in nanostructures. Nat. Nanotechnol..

[36] Na S-II, Kim S-SS, Jo J (2008). Efficient polymer solar cells with surface relief gratings fabricated by simple soft lithography. Adv. Funct. Mater..

[37] Liu Y, Tippets CA, Kirsch C, Mitran S, Samulski ET, Lopez R (2013). Balance between light trapping and charge carrier collection: electro-photonic optimization of organic photovoltaics with ridge-patterned back electrodes. J. Appl. Phys..

[38] Sha WEI, Li X, Choy WCH (2014). Breaking the space charge limit in organic solar cells by a novel plasmonic-electrical concept. Sci Rep..

[39] Choy WCH, Ren X (2016). Plasmon-electrical effects on organic solar cells by incorporation of metal nanostructures. IEEE J. Sel. Top Quantum Electron..

[40] Pivrikas A, Sarifciftci NS, Juska G, Österbacka R (2007). A review of charge transport and recombination in polymer/fullerene organic solar cells. Prog. Photovoltaics Res. Appl..

[41] Stolterfoht M, Armin A, Philippa B (2015). Photocarrier drift distance in organic solar cells and photodetectors. Sci. Rep..

[42] König TAF, Ledin PA, Kerszulis J (2014). Electrically tunable plasmonic behavior of nanocube-polymer nanomaterials induced by a redox-active electrochromic polymer. ACS Nano.

[43] Zhang Y, Cui Y, Wang W (2015). Absorption enhancement in organic solar cells with a built-in short-pitch plasmonic grating. Plasmonics.

[44] Yang HU, D’Archangel J, Sundheimer ML, Tucker E, Boreman GD, Raschke MB (2015). Optical dielectric function of silver. Phys. Rev. B..

[45] Kim JB, Guan ZL, Shu AL, Kahn A, Loo YL (2011). Annealing sequence dependent open-circuit voltage of inverted polymer solar cells attributable to interfacial chemical reaction between top electrodes and photoactive layers. Langmuir.

[46] Ghosekar IC, Patil GC (2019). Thermal stability analysis of buffered layer P3HT/P3HT:PCBM organic solar cells. IET Optoelectron..

[47] Kim H, So WW, Moon SJ (2007). The importance of post-annealing process in the device performance of poly(3-hexylthiophene): Methanofullerene polymer solar cell. Sol Energy Mater Sol Cells..

[48] Dang MT, Hirsch L, Wantz G (2011). P3HT:PCBM, best seller in polymer photovoltaic research. Adv. Mater..

[49] Chandrasekaran N, Kumar A, Thomsen L, Kabra D, McNeill CR (2021). High performance as-cast P3HT:PCBM devices: understanding the role of molecular weight in high regioregularity P3HT. Mater. Adv..

[50] Meitzner R, Faber T, Alam S (2019). Impact of P3HT materials properties and layer architecture on OPV device stability. Sol. Energy Mater. Sol. Cells..

[51] Loeza-Poot M, Méndez-Hernández J, Oviedo-Mendoza M, Mis-Fernández R, Peña JL, Hernández-Rodríguez E (2022). Effects of the processing variables on the optical properties of P3HT:PCBM absorber layer: an statical point of view. Org. Electron..

[52] Nam S, Jang J, Cha H (2012). Effects of direct solvent exposure on the nanoscale morphologies and electrical characteristics of PCBM-based transistors and photovoltaics. J. Mater. Chem..

[53] Kadem B, Hassan A, Cranton W (2016). Efficient P3HT:PCBM bulk heterojunction organic solar cells; effect of post deposition thermal treatment. J. Mater. Sci. Mater. Electron..

[54] Munshi J, Balasubramanian G (2020). Investigating blend morphology of P3HT:PCBM bulk heterojunction solar cells by classical atomistic simulations–progress and prospects. Soft. Mater..

[55] Choi JK, Jin JK, Jin ML, An CJ, Jung HT (2014). Comparison of blend morphologies of the nano-patterned photoactive films via two different techniques: thermal-assisted and solvent-assisted soft-nanoimprint lithography. RSC Adv..

[56] Rafique S, Abdullah SM, Sulaiman K, Iwamoto M (2017). Fundamentals of bulk heterojunction organic solar cells: an overview of stability/degradation issues and strategies for improvement. Renew. Sustain. Energy Rev..

[57] Sievers DW, Shrotriya V, Yang Y (2006). Modeling optical effects and thickness dependent current in polymer bulk-heterojunction solar cells. J. Appl. Phys..

[58] Lenes M, Morana M, Brabec CJ, Blom PWMM (2009). Recombination-limited photocurrents in low bandgap polymer/fullerene solar cells. Adv. Funct. Mater..

[59] Namkoong G, Kong J, Samson M, Hwang IW, Lee K (2013). Active layer thickness effect on the recombination process of PCDTBT:PC71BM organic solar cells. Org. Electron..

[60] Small CE, Tsang S-W, Chen S (2013). Loss mechanisms in thick-film low-bandgap polymer solar cells. Adv. Energy Mater..

[61] Kaienburg P, Rau U, Kirchartz T (2016). Extracting information about the electronic quality of organic solar-cell absorbers from fill factor and thickness. Phys. Rev. Appl..

[62] Udum Y, Denk P, Adam G (2014). Inverted bulk-heterojunction solar cell with cross-linked hole-blocking layer. Org. Electron Phys. Mater. Appl..

[63] Maier SA (2007). Plasmonics: fundamentals and applications.

[64] Barnes WL, Dereux A, Ebbesen TW (2003). Surface plasmon subwavelength optics. Nature.

[65] Tvingstedt K, Tang Z, Inganäs O (2012). Light trapping with total internal reflection and transparent electrodes in organic photovoltaic devices. Appl. Phys. Lett..

[66] Disney CER, Pillai S, Green MA (2017). The impact of parasitic loss on solar cells with plasmonic nano-textured rear reflectors. Sci. Rep..

[67] Zou Y, Sheng X, Xia K, Fu H, Hu J (2014). Parasitic loss suppression in photonic and plasmonic photovoltaic light trapping structures. Opt Express..

[68] Cowan SR, Wang J, Yi J, Lee YJ, Olson DC, Hsu JWP (2013). Intensity and wavelength dependence of bimolecular recombination in P3HT:PCBM solar cells: a white-light biased external quantum efficiency study. J Appl Phys..

[69] Lakhwani G, Rao A, Friend RH (2014). Bimolecular recombination in organic photovoltaics. Annu. Rev. Phys. Chem..

[70] Goodman AM, Rose A (1971). Double extraction of uniformly generated electron-hole pairs from insulators with noninjecting contacts. J. Appl. Phys..

[71] Mihailetchi VD, Wildeman J, Blom PWM (2005). Space-charge limited photocurrent. Phys. Rev. Lett..

[72] Koster LJA, Mihailetchi VD, Xie H, Blom PWM (2005). Origin of the light intensity dependence of the short-circuit current of polymer/fullerene solar cells. Appl Phys Lett..

[73] Wilken S, Sandberg OJ, Scheunemann D, Österbacka R (2020). Watching space charge build up in an organic solar cell. Sol. RRL..

