# Supplementary material for: The impact of plasmonic electrodes on the photocarrier extraction of inverted organic bulk heterojunction solar cells
Source: Appl Phys A Mater Sci Process. 2023 Mar 2;129(3):230. doi: 10.1007/s00339-023-06492-6 (PMC9977711; doi:10.1007/s00339-023-06492-6)
Supplement: Supplementary file 1 — Supplementary file1 (DOCX 1944 KB) [file 339_2023_6492_MOESM1_ESM.docx]

SUPPORTING INFORMATION

**The Impact of Plasmonic Electrodes on the Photocarrier Extraction of Inverted Organic Bulk Heterojunction Solar Cells**

*Florian Kolb*^(1)^, *Mirella El Gemayel^(1)^*, *Imran Khan*^(2)^, *Jakub Dostalek*^(2,3)^, *Roman Trattnig*^(1)^, *Christian Sommer*^(1)^, *Emil J. W. List-Kratochvil*^(4,5)^

^(1)^ Institute of Surface Technologies and Photonics, JOANNEUM RESEARCH Forschungsges. mbH, Franz-Pichler-Straße 30, 8160 Weiz, Austria

^(2)^ AIT-Austrian Institute of Technology GmbH, BioSensor Technologies, Konrad-Lorenz-Straße 24, 3430 Tulln, Austria

^(3)^ FZU-Institute of Physics, Czech Academy of Sciences, Na Slovance, Prague 182 21, Czech Republic

^(4)^ Institut für Physik, Institut für Chemie & IRIS Adlershof, Humboldt-Universität zu Berlin, Zum Großen Windkanal 2, 12489 Berlin, Germany

^(5)^ Helmholtz-Zentrum Berlin für Materialien und Energie GmbH, Hahn-Meitner-Platz 1, 14109 Berlin, Germany

E-mail: emil.list-kratochvil@hu-berlin.de

Keywords: organic solar cells, electrode patterning, optical enhancement, surface plasmons, dielectric waveguide modes, charge carrier extraction


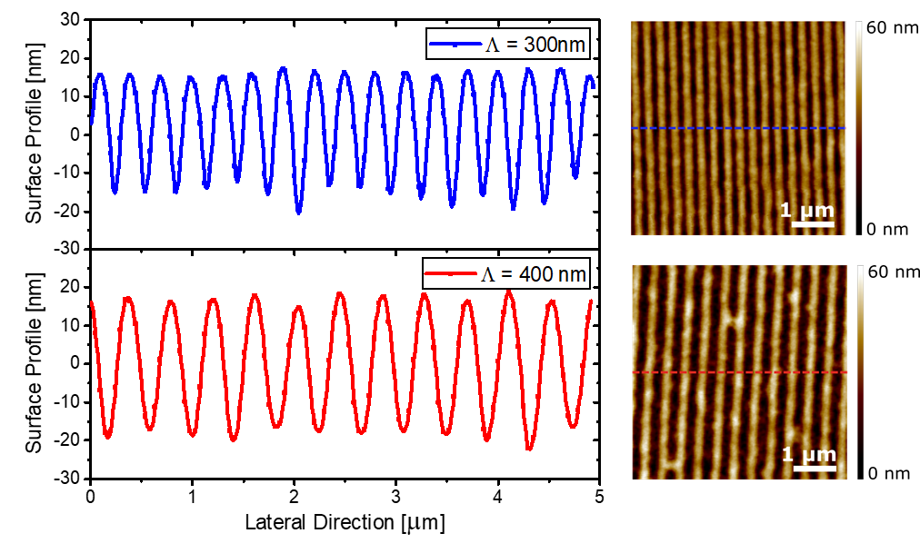


Figure S1: Surface profiles (left) and AFM height images (right) of the (linear) 2D grating profile imprinted into the active layer (P3HT:PCBM) bearing a pattern periodicity (Λ) of 300 nm (top) and 400 nm (bottom) respectively. The imprint depth (i.e. peak-to-trench distance) was about 30 nm for both applied pattern periodicities. Displayed surface profiles were taken from the location marked by the colored horizontal lines in the AFM height image.


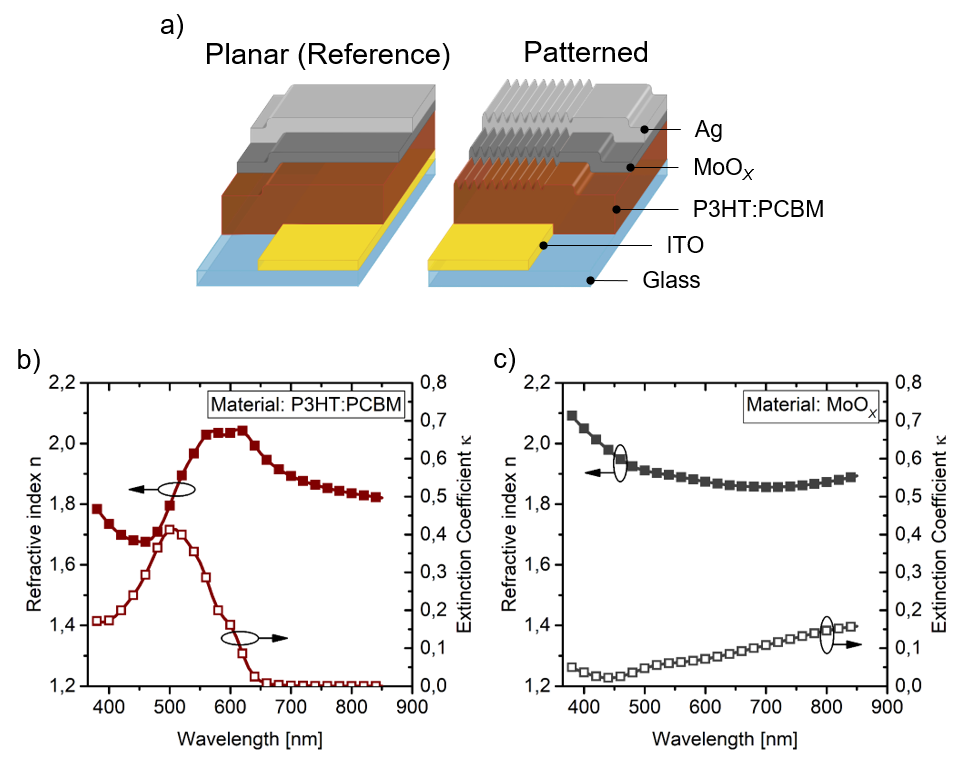


Figure S2: (a) The layer sequence utilized at the FDTD-based numerical modelling approach. Refractive index and extinction coefficient, experimentally derived through variable angle ellipsometry measurements of (b) the photoactive P3HT:PCBM blend and (c) the MoO_X_ material.

For evaluating the refractive index of the P3HT:PCBM layers, the P3HT and the PCBM compounds were blended at a ratio of 1 : 0.75 (P3HT:PCBM), dissolved in chlorobenzene and deposited via a spin-coating on a Si-Wafer bearing a oxide layer thickness of 150 nm. Similar spin-coating parameters were used as the ones employed at the solar cell fabrication (i.e. rate: 1200 s, duration: 80 s). Afterwards, deposited P3HT:PCBM layers were thermally annealed for a duration of 20 minutes at a temperature of 135°C. The solid compound to solvent ratio of investigated P3HT:PCBM layers was 10 g/l, 20 g/l and 30 g/l and a layer thickness of 80 ± 8 nm, 135 ± 7 nm and 230 ± 11 nm as observed via AFM on a scratch in the film. The P3HT:PCBM layers were investigated over a wavelength range of 300 – 1200 nm and at an angle of incidence of 65° and 70° respectively. The ellipsometry raw data obtained from the P3HT:PCBM films was analyzed through the WVASE software package (J.A. Woolam Co., Inc.). Here, the refractive index of the P3HT:PCBM material was modelled through a series of Gaussian oscillators and by applying a linearly graded index model consisting of five discrete sublayers. The refractive index of the P3HT:PCBM layer, further used at our simulations and presented in Figure S2 b, was derived by averaging the refractive indices obtained from the three respective P3HT:PCBM layer thicknesses.
To estimate the refractive index of the MoO*_X_* layer, MoO_3_ was thermally evaporated at a base pressure < 10^-6^ mbar and at a deposition rate of 0.1 Å/s (similar parameters were used at the fabrication of the solar cell devices). The MoO*_X_* layer thickness was monitored by the thin-film thickness monitor of the thermal evaporation unit and deposition was stopped reaching a layer thickness of 80 nm, 135 nm and 200 nm respectively. Ellipsometry raw data was obtained over a wavelength range of 250 – 1700 nm and at an angle of incidence of 65°, 70° and 75° respectively. The refractive index of the MoO*_X_* layers was also modelled via the WVASE software package through a set of coupled Gaussian oscillators but without adopting additional modelling approaches (e.g. a graded index model). Similar to the refractive index of the P3HT:PCBM layer, the refractive index of the MoO*_X_* material further used at the simulations and displayed in Figure S2 c, was derived by averaging the refractive indices observed at the different film thicknesses. For both P3HT:PCBM and MoO*_X_* films, the impact of the surface roughness as well as the anisotropy of the deposited films has not been specifically addressed at our modelling approach.


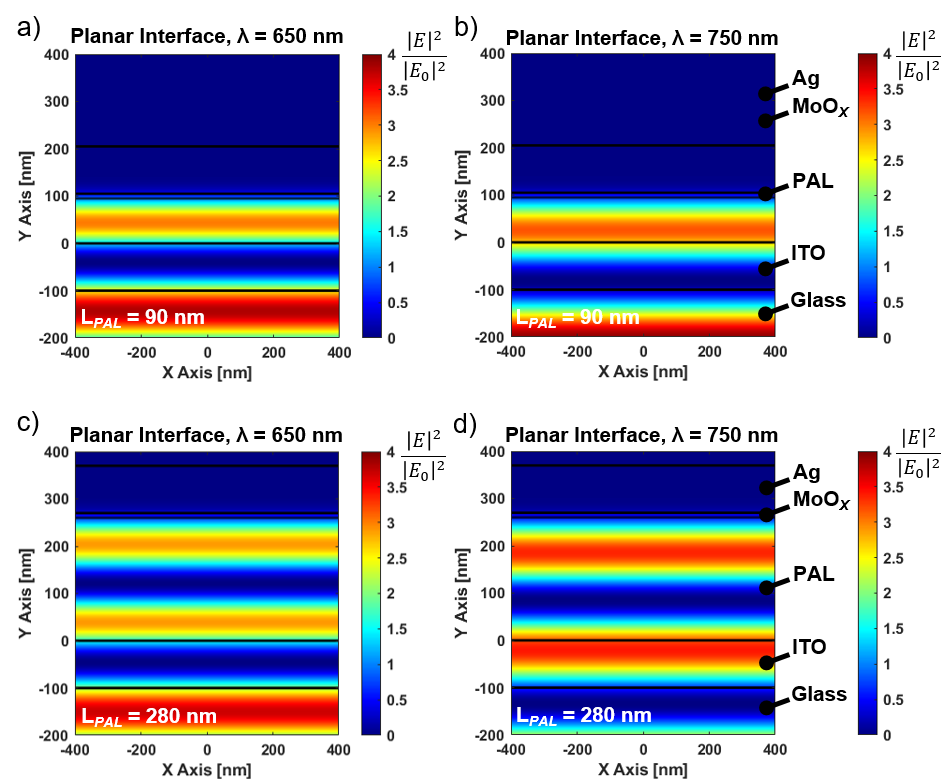


Figure S3: The spatial distribution of the optical near-field enhancements (i.e. ${\left| E \right|^{2}}/{\left| E_{0} \right|^{2}}$) observed for solar cells featuring a planar interface. Displayed spatial distributions were obtained at a wavelength of 650 nm and 750 nm for an active layer thickness (L_PAL_) of (a,b) 90 nm and (c,d) 280 nm respectively. Compared to the optical near-field enhancements observed for patterned devices, the near-field enhancements are quite homogeneously distributed at planar devices, reflecting the plane wave characteristic of the light source and the angle of incidence (ϕ = 0) used at the simulations. Notably, the magnitude of the near-field optical enhancements of planar devices is significantly lower than the magnitude of the optical near-field enhancements observed at patterned solar cells.


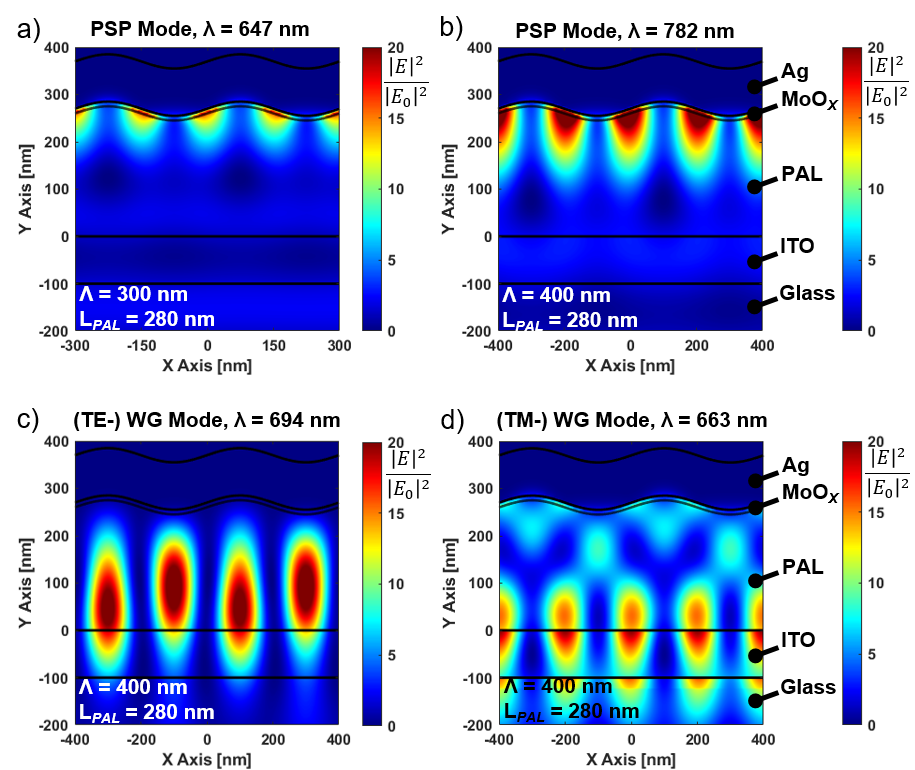


Figure S4: Spatial distribution of the optical near-field enhancements (i.e. ${\left| E \right|^{2}}/{\left| E_{0} \right|^{2}}$) observed at an active layer thickness (L_PAL_) of 280 nm for patterned devices bearing a pattern periodicity (Λ) of (a) 300 nm and (b - d) 400 nm respectively. Presented near-field enhancements were particularly obtained at wavelengths where light resonantly couples to propagating surface plasmon (PSP) and dielectric waveguide (WG) modes. Since sinusoidal 2D grating profiles were used for patterning the active layer/back electrode interface, the occurrence of surface plasmon and waveguide modes depends on the polarization state of incident light: PSP modes are observed for TM-polarized light only whereas WG modes are observed for both TM- and TE- polarized light.


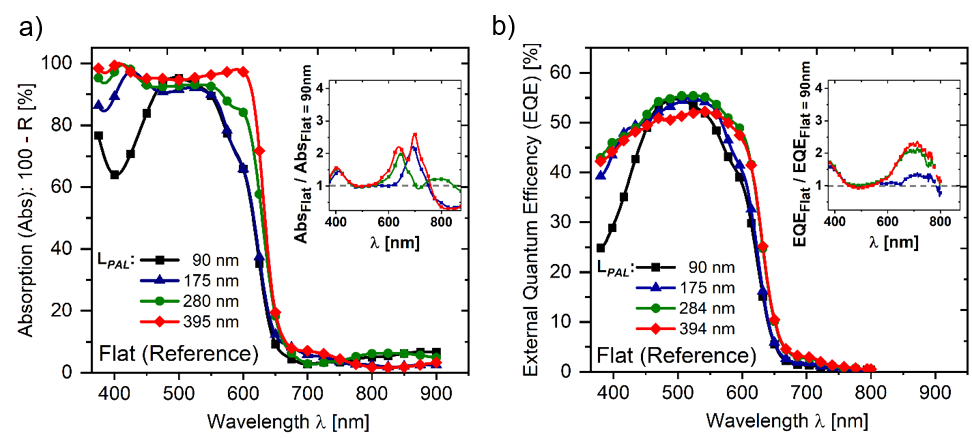


Figure S5: (a) Simulated absorption characteristic of planar (i.e. flat) solar cells for an active layer thickness (L_PAL_) that was gradually increased from 90 nm (reference thickness) to 390 nm. (b) Spectral dependent external quantum efficiency characteristic of fabricated planar solar cells. The relative enhancement of the absorption and the EQE characteristic when increasing L_PAL_ from our reference thickness is displayed at the inset graphs in (a) and (b) respectively.


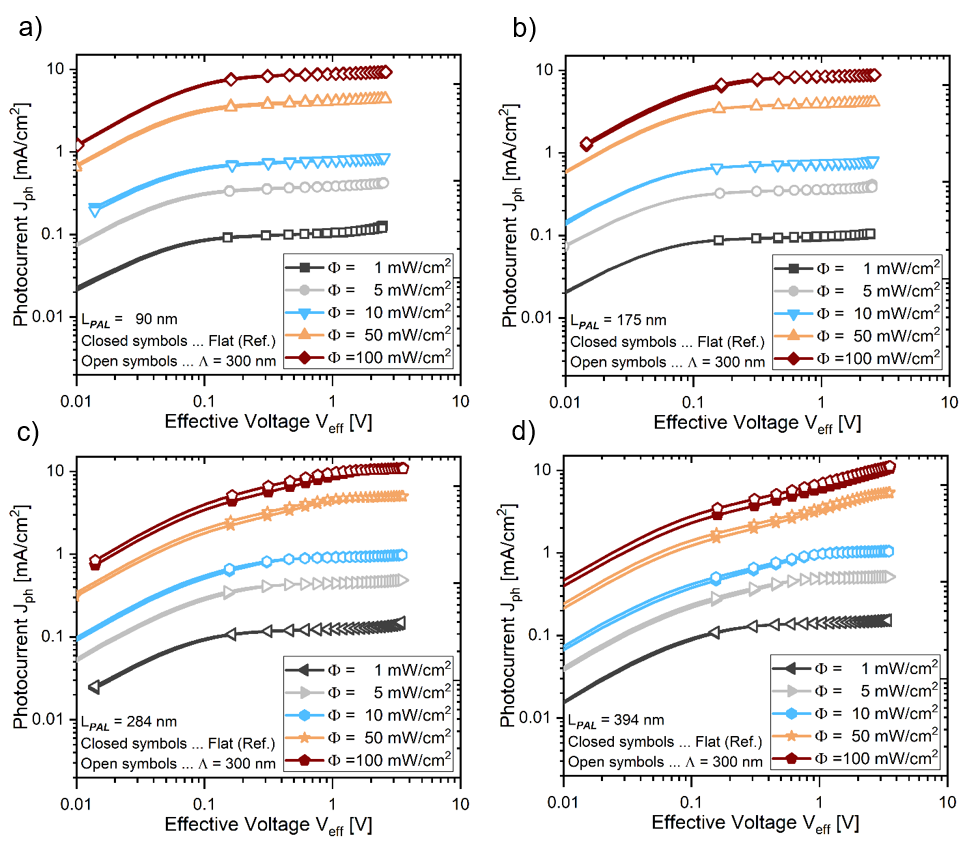


Figure S6: The voltage dependent photocurrent characteristic of planar and Λ = 300 nm patterned solar cells, observed for irradiance levels (Φ) gradually varied in the range between 1 – 100 mW/cm^2^ and for an active layer thickness (L_PAL_) of (a) 90 nm, (b) 175 nm, (c) 284 nm and (d) 395 nm respectively. Irradiance levels of the incident unpolarized light were varied through a set of neutral density filters.


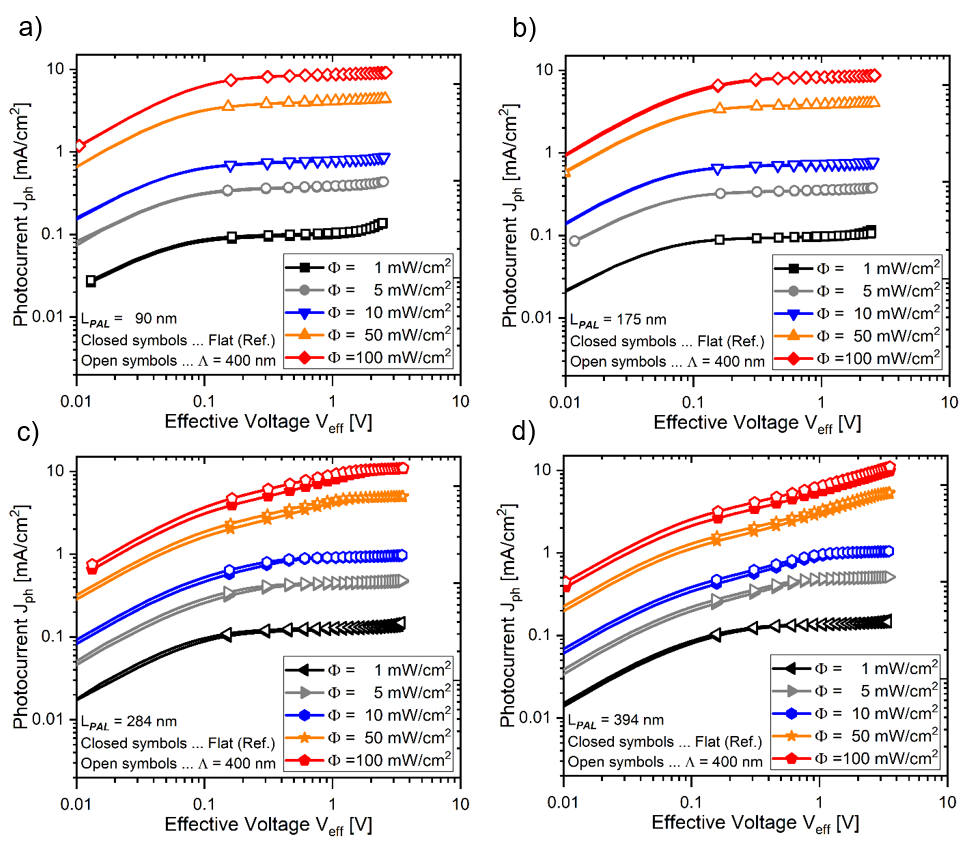


Figure S7: Comparison of the voltage dependent photocurrent characteristic of planar and Λ = 400 nm patterned solar cells, observed for irradiance levels (Φ) in the range between 1 – 100 mW/cm^2^ and for an active layer thickness (L_PAL_$)$ of (a) 90 nm, (b) 175 nm, (c) 284 nm and (d) 395 nm respectively. Irradiance variation of the incident unpolarized light was realized through a set of neutral density filters.
